# Novel Reassortant Influenza A(H1N2) Virus Derived from A(H1N1)pdm09 Virus Isolated from Swine, Japan, 2012

**DOI:** 10.3201/eid1912.120944

**Published:** 2013-12

**Authors:** Miho Kobayashi, Ikuyo Takayama, Tsutomu Kageyama, Hiroyuki Tsukagoshi, Mika Saitoh, Taisei Ishioka, Yoko Yokota, Hirokazu Kimura, Masato Tashiro, Kunihisa Kozawa

**Affiliations:** Gunma Prefectural Institute of Public Health and Environmental Sciences, Maebashi-shi, Gunma, Japan (M. Kobayashi, H. Tsukagoshi, M. Saitoh, T. Ishioka, Y. Yokota, K. Kozawa);; National Institute of Infectious Diseases, Musashimurayama-shi, Tokyo, Japan (I.Takayama, T. Kageyama, H. Kimura, M. Tashiro)

**Keywords:** Swine, influenza A (H1N2), virus, reassortant viruses, H1N1 subtype

## Abstract

We isolated a novel influenza virus A(H1N2) strain from a pig on January 13, 2012, in Gunma Prefecture, Japan. Phylogenetic analysis showed that the strain was a novel type of double-reassortant virus derived from the swine influenza virus strains H1N1pdm09 and H1N2, which were prevalent in Gunma at that time.

Influenza A viruses can be transmitted between humans, swine, and birds; virus subtypes have the potential to reassort and generate new viruses by cross-breeding in the various hosts (*1*). For example, influenza A subtype H1N1 viruses reassorted in swine, and the resulting swine influenza viruses (SIVs) were transmitted to humans. The reassorted combinations have resulted in pandemic viruses as well as low-pathogenicity viruses with low transmissibility among humans. Similarly, seasonal human subtypes of influenza are transmissible to swine (*2*). In 2009, a novel strain of the H1N1 SIV subtype emerged and was associated with a pandemic (*3*,*4*). The virus, later termed influenza A(H1N1)pdm09, hereafter referred to as pH1N1, was confirmed as a reassortant virus resulting from cross-breeding of a European avian subtype H1N1 virus and a North American triple reassortant virus (*5*). Subsequently, other strains reassorted from the pH1N1 virus (*6*–*8*). We report on an isolated new reassortant H1N2 SIV derived from the pH1N1 virus and SIVs originating in Japan. 

## The Study

We collected 109 nasal swab samples from pigs for swine influenza surveillance during November 2011–February 2012. Nasal swab samples were collected from healthy pigs, 6 months of age, at an abattoir in Gunma Prefecture, Japan. All samples were inoculated onto MDCK cells (*9*). All cell culture supernatants were tested by using a hemagglutination assay of a 0.7% solution of guinea pig erythrocytes (*9*). To determine the subtype of the isolate, a hemagglutination inhibition assay was performed by using ferret antiserum for A/California/07/2009 [A(H1N1)pdm09], A/Victoria/210/2009 [A(H3N2)], B/Bangladesh/3333/2007 [B/Yamagata-lineage], and B/Brisbane/60/2008 [B/Victoria-lineage] (*9*). One strain of influenza A virus, designated A/swine/Gunma/1/2012, was isolated from the samples. 

For full genome sequencing of the influenza A/swine/Gunma/1/2012 strain, we conducted reverse transcription PCR (*10*). Segment-specific primers used for amplification and sequencing are shown in Technical Appendix Figure, panel A. Phylogenetic analysis of the nucleotide sequences was conducted by using MEGA version 5 software (www.megasoftware.net) and Tree Explorer version 2.12 (http://en.bio-soft.net/tree/TreeExplorer.html) (*11*). Evolutionary distances were estimated according to the Kimura 2-parameter method (*12*). The phylogenetic trees of hemagglutinin (HA) and neuraminidase (NA) genes were constructed by using the neighbor-joining method (*13*). In addition, phylogenetic trees based on the matrix protein, nucleoprotein genes, nonstructural protein, polymerase acid, polymerase basic 1, and polymerase basic 2 were constructed by using the neighbor-joining method. The reliability of the trees was estimated with 1,000 bootstrap replications. GenBank accession numbers assigned to the gene sequences of the analyzed strain are the following: polymerase basic 2 (AB731582), polymerase basic 1 (AB731583), polymerase acid (AB731584), HA (AB731585), nucleoprotein (AB731586), NA (AB731587), matrix protein (AB731588), and nonstructural protein (AB731589).

Phylogenetic trees based on HA and NA gene sequences are shown in the Figure, panels A and B. The identities of the nucleotide sequences of each gene are shown in the Table. The A/swine/Gunma/1/2012 strain was confirmed as a strain of pH1N1 virus Figure, panel A). NA gene sequences showed that the virus was located within clusters of swine-type viruses documented in Japan as the representative strains, such as A/swine/Ehime/1/1980 (Figure, panel B). The sequence identity of the NA gene between the A/swine/Gunma/1/2012 strain and other Japanese H1N2 SIV strains ranged from 85.0 to 97.5%. The identities of other genes between the A/swine/Gunma/1/2012 strain and pH1N1 virus vaccine strain (A/California/07/2009) were highly homologous (>90%; Table). These results suggest that the A/swine/Gunma/1/2012 strain was a new reassortant of the H1N2 SIV subtype derived from the pH1N1 virus.

**Figure:**
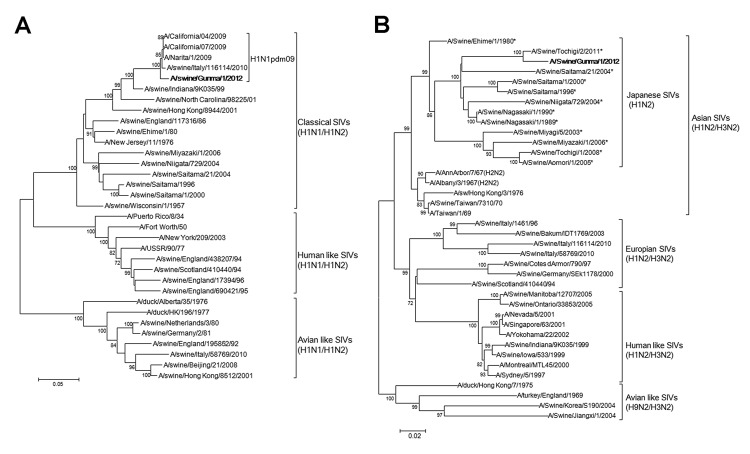
Phylogenetic tree based on the nucleotide sequences of hemagglutinin (A) and neuraminidase (B) genes of A/swine/Gunma/1/2012, a novel H1N2 swine influenza virus (SIV) strain. Distance was calculated according to the Kimura 2-parameter method; the trees were constructed by using the neighbor-joining method with labeling of the branches showing at least 70% bootstrap support. Boldface text indicates the novel strain reassorted from strains of the SIV H1N2 subtype. Asterisks indicate reference strains compared with A/Swine/Gunma/1/2012 used to calculate the identity of neuraminidase gene. Scale bars indicate nucleotide substitutions per site.

**Table:** Sequence identity of each gene of influenza strain A/swine/Gunma/1/2012, reassorted from influenza A(H1N1)pdm09 and A/California/07/2009*

Gene	Identity (%)
PB2	98.9
PB1	98.7
rPA	98.7
HA	98.4
NP	98.7
MP	99.3
NS	99.3

*PB, polymerase basic; PA, polymerase acid; HA, hemagglutinin; NP, nucleoprotein; MP, matrix protein; NS, nonstructural protein.

We isolated 1 strain in this study. The samples (109 nasal swabs) were collected from different pig farms ≈60 km apart. The epidemiologic association may be low among the samples, because the quarantine inspection system is well established in Japan. All samples were collected from pigs 6 months of age; therefore, the potential for infection with the virus could have been low. Additional and larger studies investigating the emergence of the parent virus of the strain may be needed.

## Conclusions

Vijaykrisna et al. found a new reassortant virus among avian-type, swine-type, and pH1N1 viruses (*6*). In addition, Monero et al. reported a new reassortant virus between SIV, identified in Italy, and pH1N1 viruses (*7*). Thus, pH1N1 virus and other types of influenza viruses can be reassorted. However, to our knowledge, reassortant H1N2 SIV strains derived from pH1N1 virus in Japan have not been identified before this report. Although the transmission of SIVs to humans has been reported sporadically, the infectious nature of this reassortant H1N2 strain among humans is unknown. The emergence of a novel H1N2 SIV strain raises further concerns about whether the virus will generate further genetic reassortments and gain virulence. Systematic influenza virus surveillance in pigs and humans should be considered.

Technical Appendix Genome Amplification, Sequencing, and Phylogeny of Novel Reassortant Influenza A(H1N2) Virus
