# Supplementary material for: Novel Reassortant Influenza A(H1N2) Virus Derived from A(H1N1)pdm09 Virus Isolated from Swine, Japan, 2012
Source: Emerg Infect Dis. 2013 Dec;19(12):1972–4. doi: 10.3201/eid1912.120944 (PMC3840884; doi:10.3201/eid1912.120944)
Supplement: Technical Appendix — Genome Amplification, Sequencing, and Phylogeny of Novel Reassortant Influenza A(H1N2) Virus [file 12-0944-Techapp-s1.pdf]

Article DOI: http://dx.doi.org/10.3201/eid1912.120944

Novel Reassortant Influenza A(H1N2) Virus Derived from A(H1N1)pdm09 Virus Isolated from Swine, Japan, 2012

Technical Appendix

Genome Amplification, Sequencing, and Phylogeny of Novel Reassortant Influenza A(H1N2) Virus

Technical Appendix Table. Primers for amplification and sequencing of the full genome of A/swine/Gunma/1/2012, a novel swine influenza A(H1N2) virus strain, Japan, 2012

Gene	Primer	Sequence (5′→3′)
Amplification		
HA	5+HA-1	CAGGGAGCAAAAGCAGGGG
	11+NS-890R	CGTCTCGTATTAGTAGAAACAAGGGTGTTTT
NA	7+NA-1	CTCAGGGAGCAAAAGCAGGAGT
	12+NA-1413R	TGGTCTCGTATTAGTAGAAACAAGGAGTTTTTT
PB2	PB2-F	AGCAAAAGCAGGTCAAWTATTC
	PB2-R	AGTAGAAACAAGGTCGTTTTTAAA
PB1	PB1-1	AGCAAAAGCAGGCAAACCATT
	PB1-R	AGTAGAAACAAGGCATTTTTTCA
PA	PA-1-26F	AGCRAAAGCAGGTACTGATYCRAAAT
	PA-2228R	AGTAGAAACAAGGTACTTTTTTGGACAG
NP	NP-F	AGCAAAAGCAGGGTARATAATCACTC
	Bm-NP-1565R	AGTAGAAACAAGGGTATTTTT
MP	MP-F	AGCAAAAGCAGGTAGATRTTKAAAG
	MP-R	AGTAGAAACAAGGTAGTTTTTTACTC
NS	NS-F	AGCAAAAGCAGGGTGACAAA
	NS-890R	AGTAGAAACAAGGGTGTTTTTTATCA
Sequencing		
HA	5+HA-1	CAGGGAGCAAAAGCAGGGG
	swineH1-56-76F	CATTATGTATAGGTTATCATG
	swineH1-366-385F	GAGCTCAGTGTCATCATTTG
	swineH1-596-578R	GGATGGTGAATGCCCCATA
	swineH1-768-788F	AATAACATTCGAAGCAACTGG
	swineH1-1106-1087R	TGATAACCGTACCATCCATC
	H1N1pdmHA-1300-1320F	GACATTTGGACTTACAATGCC
NA	7+NA-1	CTCAGGGAGCAAAAGCAGGAGT
	N2-548R	TGACAACTTGAGCTGGACCA
	N2-1062R	AAGGCCCAGCCTTTCACYCC
	N2-902F	TGGAAGGGCTCYAATAGGCC
	12+NA-1413R	TGGTCTCGTATTAGTAGAAACAAGGAGTTTTTT
PB2	PB2-F	AGCAAAAGCAGGTCAAWTATTC
	PB2-599R	ATGTATGCCACCATYAAGGG
	H1N1pdmPB2-280-300R	TTCCACCATGTTACGGCCAGA
	H5PB2-1241R	TATCATGCAATCCTCCTGTG
	H5PB2-1071F	CCTCCAAACATTGAAAATAAGAG
	PB2-R	AGTAGAAACAAGGTCGTTTTTAAA
	H1N1pdmPB2-1650-1670F	CAATGGATAATCAGGAACTG
PB1	H1N1pdmPB1-290-310R	CAAATATTCCTGGGTGGGATT
	PB1-1	AGCAAAAGCAGGCAAACCATT
	PB1-1240R	AACATGCCCATCATCATTCC
	PB1-1334R	GATTGGAGTCGATCCCACCA
	PB1-1121F	CACAAATACCAGCAGAAATGC
	PB1-R	AGTAGAAACAAGGCATTTTTTCA

Gene	Primer	Sequence (5′→3′)
	PB1-1651F	ACAGCTCAGATGGCTCTTCA
PA	PA-230-249R	TTCAATTATCTCAAATCGGT
	H5PA-583R	TGACGAAAGGAATCCCATAG
	H5PA-686R	TTCAAGGCTGGAGAAGTTCG
	PA-940Rv2	TCTTCATGCATTTGATTGCATC
	PA-1-26F	AGCRAAAGCAGGTACTGATYCRAAAT
	H5PA-583F	CTATGGGATTCCTTTCGTCA
	H5PA-1818R	TCTCTTTGACAGAAGACTCG
	PA-940F	GATGCAATCAAATGCATGAAGA
	H5PA-1147F	GCACCAGAGAAAGTAGACTT
	PA-2228R	AGTAGAAACAAGGTACTTTTTTGGACAG
	H5PA-1619F	TGGAGCCACACAAGTGGGAA
	H5PA-F1792	GCCGAGTCTTCTGTCAAAGA
NP	H1N1pdmNP-500-520R	TGGGAAGTGTTGACCCTTGCA
	NP-F	AGCAAAAGCAGGGTARATAATCACTC
	H5NP-1075R	GTCCCTCTGATGAAACTTGA
	H5NP-451F	CTGATGATATGGCATTCCAA
	NP-1565R	AGTAGAAACAAGGGTATTTTTCTT
	H5NP-451F	CTGATGATATGGCATTCCAA
	H1N1pdmNP-1000-1020F	CACTCTGCTGCATTTGAAGAT
MP	MP-501r2	CGATGCTGTGAATCTGCAAT
	MP-R	AGTAGAAACAAGGTAGTTTTTTACTC
	MP-F	AGCAAAAGCAGGTAGATRTTKAAAG
	H5MP-501F	ATTGCAGATTCACAGCATCG
NS	NS-F	AGCAAAAGCAGGGTGACAAA
	H5NS-535R	GTGTTATCATTCCATTCAAGTCC
	NS-890R	AGTAGAAACAAGGGTGTTTTTTATCA
	H1N1pdmNS-450-470F	CACTGAGGAGGGAGCAATAGT

Technical Appendix Figure (following pages). Phylogenetic analysis of the A/swine/Gunma/1/2012 strain of the influenza A(H1N1)pdm09 virus isolated from swine. Phylogenetic trees based on A) the matrix protein (MP), B) nucleoprotein (NP), C) nonstructural protein (NS), D) polymerase acid (PA), E) polymerase basic 1 (PB1), and F) polymerase basic 2 (PB2) genes were constructed by using the neighbor joining method. Bold text represents novel H1N2 swine influenza virus (SIV) isolated in Japan during January 2012. Numbers along branches represent estimates made with 1,000 bootstrap replications to demonstrate the reliability of the trees. Scale bars represent nucleotide substitutions by site.

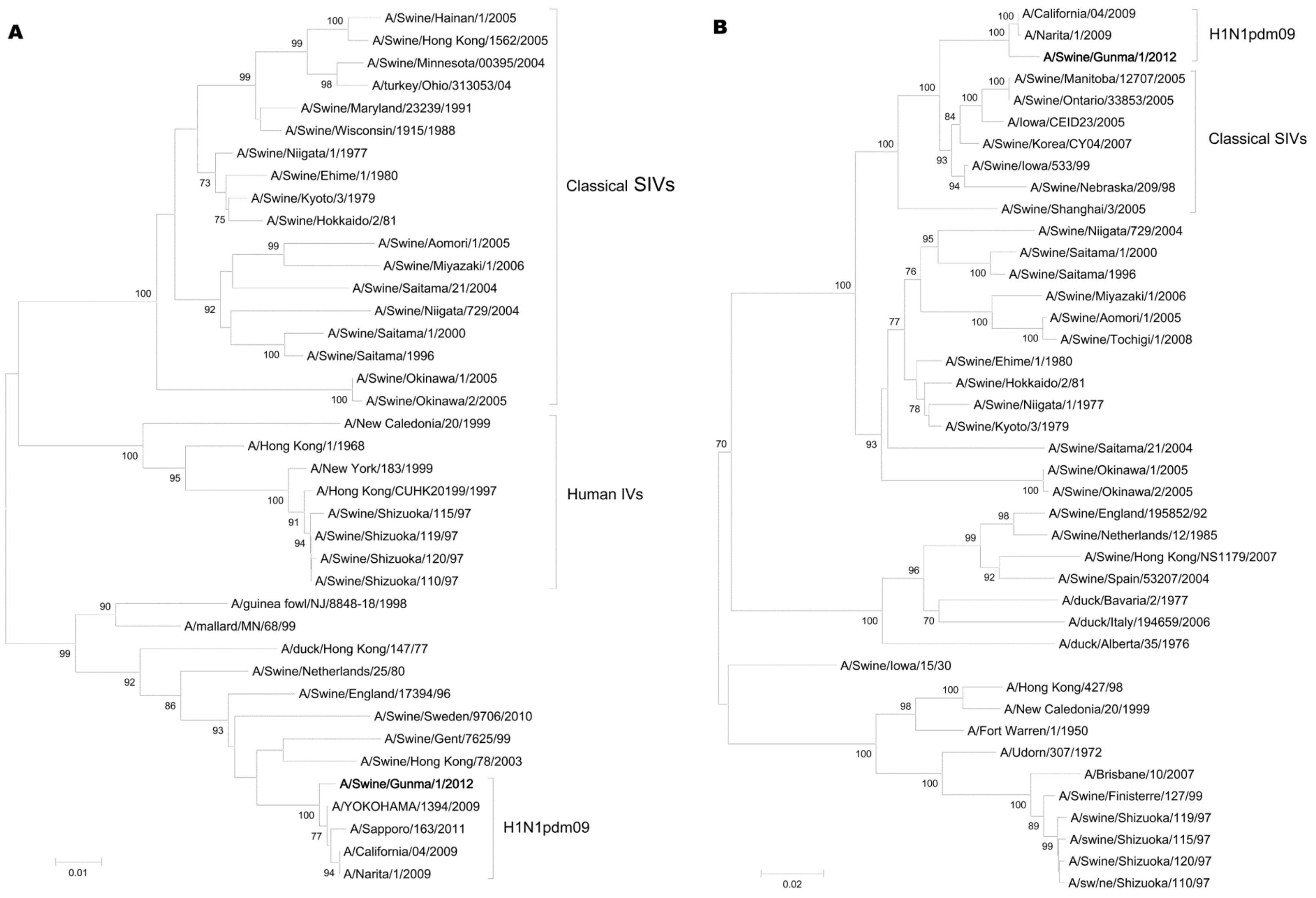
A
A/Swine/Hainan/1/2005
A/Swine/Hong Kong/1562/2005
A/Swine/Minnesota/00395/2004
A/turkey/Ohio/313053/04
A/Swine/Maryland/23239/1991
A/Swine/Wisconsin/1915/1988
A/Swine/Niigata/1/1977
A/Swine/Ehime/1/1980
A/Swine/Kyoto/3/1979
A/Swine/Hokkaido/2/81
A/Swine/Aomori/1/2005
A/Swine/Miyazaki/1/2006
A/Swine/Saitama/21/2004
A/Swine/Niigata/729/2004
A/Swine/Saitama/1/2000
A/Swine/Saitama/1996
A/Swine/Okinawa/1/2005
A/Swine/Okinawa/2/2005
Classical SIVs
A/New Caledonia/20/1999
A/Hong Kong/1/1968
A/New York/183/1999
A/Hong Kong/CUHK20199/1997
A/Swine/Shizuoka/115/97
A/Swine/Shizuoka/119/97
A/Swine/Shizuoka/120/97
A/Swine/Shizuoka/110/97
Human IVs
A/guinea fowl/NJ/8848-18/1998
A/mallard/MN/68/99
A/duck/Hong Kong/147/77
A/Swine/Netherlands/25/80
A/Swine/England/17394/96
A/Swine/Sweden/9706/2010
A/Swine/Gent/7625/99
A/Swine/Hong Kong/78/2003
A/Swine/Gunma/1/2012
A/YOKOHAMA/1394/2009
A/Sapporo/163/2011
A/California/04/2009
A/Narita/1/2009
H1N1pdm09
0.01
B
A/California/04/2009
A/Narita/1/2009
A/Swine/Gunma/1/2012
H1N1pdm09
A/Swine/Manitoba/12707/2005
A/Swine/Ontario/33853/2005
A/Iowa/CEID23/2005
A/Swine/Korea/CY04/2007
A/Swine/Iowa/533/99
A/Swine/Nebraska/209/98
A/Swine/Shanghai/3/2005
Classical SIVs
A/Swine/Niigata/729/2004
A/Swine/Saitama/1/2000
A/Swine/Saitama/1996
A/Swine/Miyazaki/1/2006
A/Swine/Aomori/1/2005
A/Swine/Tochigi/1/2008
A/Swine/Ehime/1/1980
A/Swine/Hokkaido/2/81
A/Swine/Niigata/1/1977
A/Swine/Kyoto/3/1979
A/Swine/Saitama/21/2004
A/Swine/Okinawa/1/2005
A/Swine/Okinawa/2/2005
A/Swine/England/195852/92
A/Swine/Netherlands/12/1985
A/Swine/Hong Kong/NS1179/2007
A/Swine/Spain/53207/2004
A/duck/Bavaria/2/1977
A/duck/Italy/194659/2006
A/duck/Alberta/35/1976
A/Swine/Iowa/15/30
A/Hong Kong/427/98
A/New Caledonia/20/1999
A/Fort Warren/1/1950
A/Udorn/307/1972
A/Brisbane/10/2007
A/Swine/Finisterre/127/99
A/swine/Shizuoka/119/97
A/swine/Shizuoka/115/97
A/Swine/Shizuoka/120/97
A/sw/ne/Shizuoka/110/97
0.02

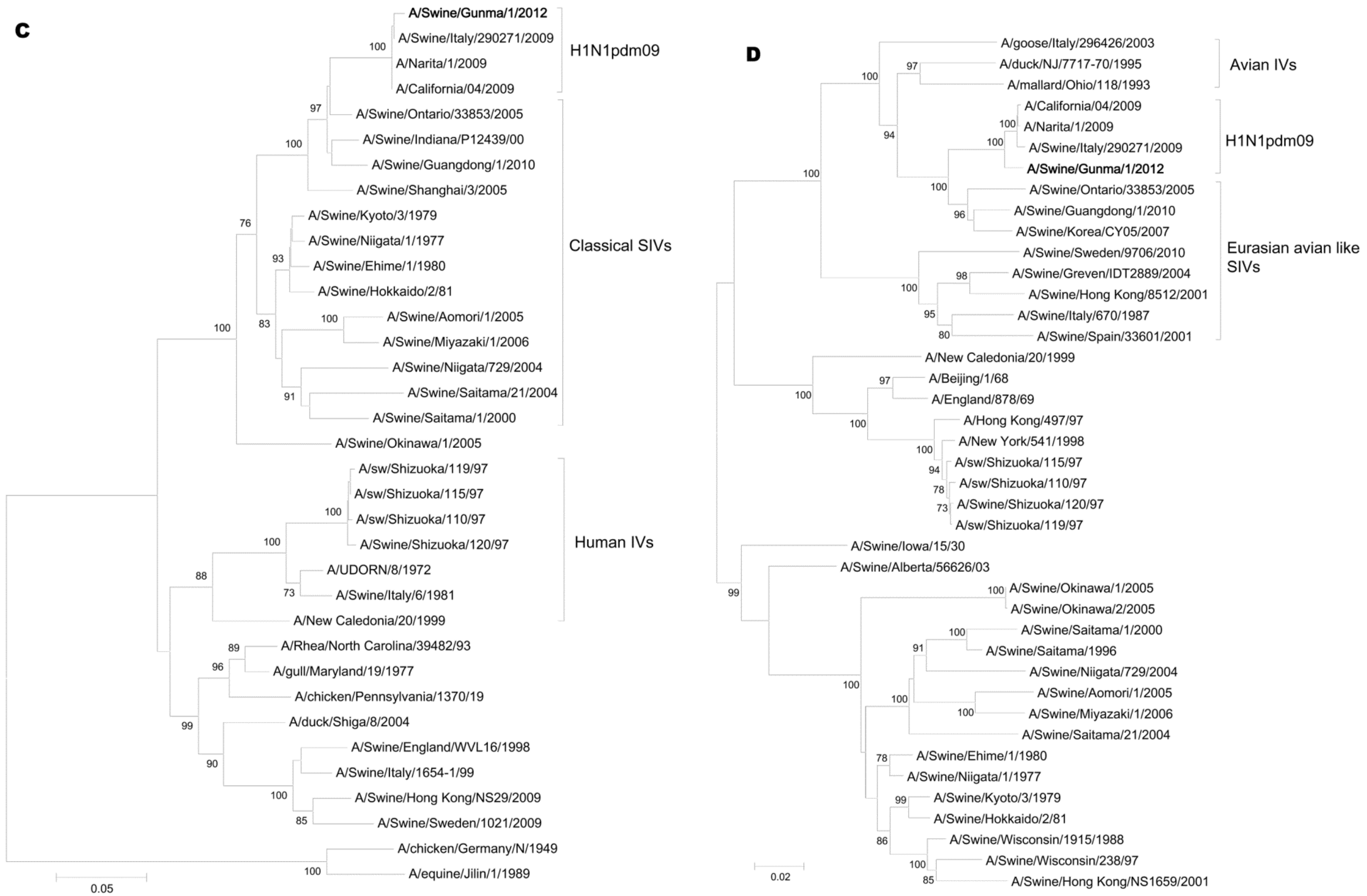
C
A/Swine/Gunma/1/2012
A/Swine/Italy/290271/2009
A/Narita/1/2009
A/California/04/2009
H1N1pdm09
A/Swine/Ontario/33853/2005
A/Swine/Indiana/P12439/00
A/Swine/Guangdong/1/2010
A/Swine/Shanghai/3/2005
A/Swine/Kyoto/3/1979
A/Swine/Niigata/1/1977
A/Swine/Ehime/1/1980
A/Swine/Hokkaido/2/81
A/Swine/Aomori/1/2005
A/Swine/Miyazaki/1/2006
A/Swine/Niigata/729/2004
A/Swine/Saitama/21/2004
A/Swine/Saitama/1/2000
Classical SIVs
A/Swine/Okinawa/1/2005
A/sw/Shizuoka/119/97
A/sw/Shizuoka/115/97
A/sw/Shizuoka/110/97
A/Swine/Shizuoka/120/97
A/UDORN/8/1972
A/Swine/Italy/6/1981
A/New Caledonia/20/1999
Human IVs
A/Rhea/North Carolina/39482/93
A/gull/Maryland/19/1977
A/chicken/Pennsylvania/1370/19
A/duck/Shiga/8/2004
A/Swine/England/WVL16/1998
A/Swine/Italy/1654-1/99
A/Swine/Hong Kong/NS29/2009
A/Swine/Sweden/1021/2009
A/chicken/Germany/N/1949
A/equine/Jilin/1/1989
0.05
D
A/goose/Italy/296426/2003
A/duck/NJ/7717-70/1995
A/mallard/Ohio/118/1993
Avian IVs
A/California/04/2009
A/Narita/1/2009
A/Swine/Italy/290271/2009
A/Swine/Gunma/1/2012
H1N1pdm09
A/Swine/Ontario/33853/2005
A/Swine/Guangdong/1/2010
A/Swine/Korea/CY05/2007
A/Swine/Sweden/9706/2010
A/Swine/Greven/IDT2889/2004
A/Swine/Hong Kong/8512/2001
A/Swine/Italy/670/1987
A/Swine/Spain/33601/2001
Eurasian avian like SIVs
A/New Caledonia/20/1999
A/Beijing/1/68
A/England/878/69
A/Hong Kong/497/97
A/New York/541/1998
A/sw/Shizuoka/115/97
A/sw/Shizuoka/110/97
A/Swine/Shizuoka/120/97
A/sw/Shizuoka/119/97
A/Swine/Iowa/15/30
A/Swine/Alberta/56626/03
A/Swine/Okinawa/1/2005
A/Swine/Okinawa/2/2005
A/Swine/Saitama/1/2000
A/Swine/Saitama/1996
A/Swine/Niigata/729/2004
A/Swine/Aomori/1/2005
A/Swine/Miyazaki/1/2006
A/Swine/Saitama/21/2004
A/Swine/Ehime/1/1980
A/Swine/Niigata/1/1977
A/Swine/Kyoto/3/1979
A/Swine/Hokkaido/2/81
A/Swine/Wisconsin/1915/1988
A/Swine/Wisconsin/238/97
A/Swine/Hong Kong/NS1659/2001
0.02

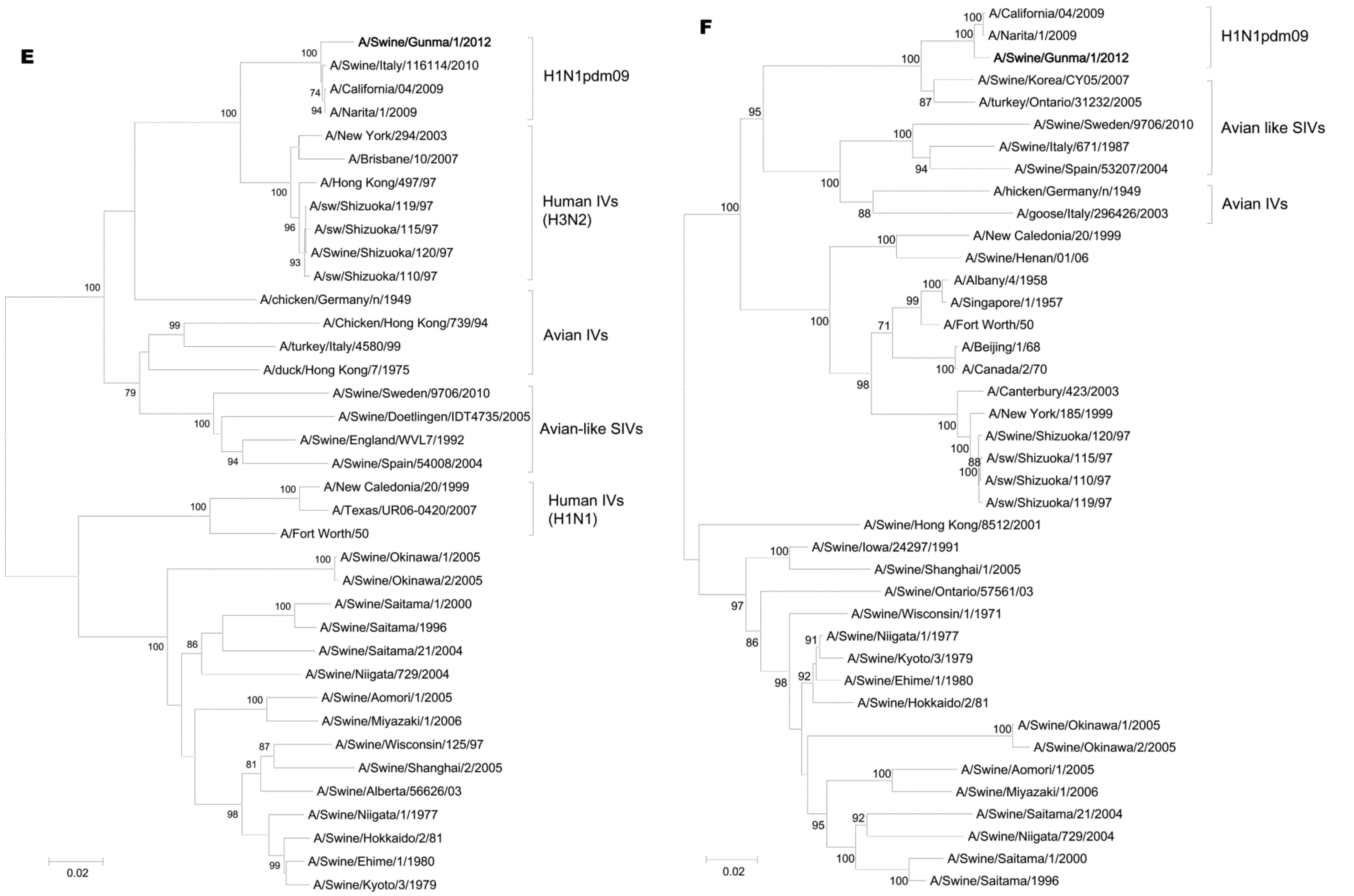
E
A/Swine/Gunma/1/2012
A/Swine/Italy/116114/2010
A/California/04/2009
A/Narita/1/2009
H1N1pdm09
A/New York/294/2003
A/Brisbane/10/2007
A/Hong Kong/497/97
A/sw/Shizuoka/119/97
A/sw/Shizuoka/115/97
A/Swine/Shizuoka/120/97
A/sw/Shizuoka/110/97
Human IVs (H3N2)
A/chicken/Germany/n/1949
A/Chicken/Hong Kong/739/94
A/turkey/Italy/4580/99
A/duck/Hong Kong/7/1975
Avian IVs
A/Swine/Sweden/9706/2010
A/Swine/Doetlingen/IDT4735/2005
A/Swine/England/WVL7/1992
A/Swine/Spain/54008/2004
Avian-like SIVs
A/New Caledonia/20/1999
A/Texas/UR06-0420/2007
A/Fort Worth/50
Human IVs (H1N1)
A/Swine/Okinawa/1/2005
A/Swine/Okinawa/2/2005
A/Swine/Saitama/1/2000
A/Swine/Saitama/1996
A/Swine/Saitama/21/2004
A/Swine/Niigata/729/2004
A/Swine/Aomori/1/2005
A/Swine/Miyazaki/1/2006
A/Swine/Wisconsin/125/97
A/Swine/Shanghai/2/2005
A/Swine/Alberta/56626/03
A/Swine/Niigata/1/1977
A/Swine/Hokkaido/2/81
A/Swine/Ehime/1/1980
A/Swine/Kyoto/3/1979
0.02
F
A/California/04/2009
A/Narita/1/2009
A/Swine/Gunma/1/2012
H1N1pdm09
A/Swine/Korea/CY05/2007
A/turkey/Ontario/31232/2005
A/Swine/Sweden/9706/2010
A/Swine/Italy/671/1987
A/Swine/Spain/53207/2004
Avian like SIVs
A/hicken/Germany/n/1949
A/goose/Italy/296426/2003
Avian IVs
A/New Caledonia/20/1999
A/Swine/Henan/01/06
A/Albany/4/1958
A/Singapore/1/1957
A/Fort Worth/50
A/Beijing/1/68
A/Canada/2/70
A/Canterbury/423/2003
A/New York/185/1999
A/Swine/Shizuoka/120/97
A/sw/Shizuoka/115/97
A/sw/Shizuoka/110/97
A/sw/Shizuoka/119/97
A/Swine/Hong Kong/8512/2001
A/Swine/Iowa/24297/1991
A/Swine/Shanghai/1/2005
A/Swine/Ontario/57561/03
A/Swine/Wisconsin/1/1971
A/Swine/Niigata/1/1977
A/Swine/Kyoto/3/1979
A/Swine/Ehime/1/1980
A/Swine/Hokkaido/2/81
A/Swine/Okinawa/1/2005
A/Swine/Okinawa/2/2005
A/Swine/Aomori/1/2005
A/Swine/Miyazaki/1/2006
A/Swine/Saitama/21/2004
A/Swine/Niigata/729/2004
A/Swine/Saitama/1/2000
A/Swine/Saitama/1996
0.02
